# Circulating Strains of *Brucella abortus* in Cattle in Santo Domingo De Los Tsáchilas Province – Ecuador

**DOI:** 10.3389/fpubh.2015.00045

**Published:** 2015-03-10

**Authors:** Richar Ivan Rodríguez-Hidalgo, Javier Contreras-Zamora, Washington Benitez Ortiz, Karina Guerrero-Viracocha, Holger Salcan-Guaman, Elizabeth Minda, Lenin Ron Garrido

**Affiliations:** ^1^Facultad de Medicina Veterinaria y Zootecnia, Universidad Central del Ecuador (UC), Quito, Ecuador; ^2^Centro Internacional de Zoonosis, Universidad Central del Ecuador (UC), Quito, Ecuador; ^3^Dirección General de Postgrado, Universidad Tecnológica Equinoccial, Santo Domingo de los Tsáchilas, Ecuador; ^4^Departamento de Ciencias de la Vida y la Agricultura, Universidad de las Fuerzas Armadas – ESPE, Carrera de Ingeniería en Ciencias Agropecuarias, Sangolqui, Ecuador; ^5^Facultad de Ciencias Agrícolas, Universidad Central del Ecuador (UC), Quito, Ecuador

**Keywords:** *Bovine brucellosis*, *Brucella abortus*, Ecuador, *Brucella abortus* biotype 1, *Brucella abortus* biotype 4, VNTR

## Abstract

The Province of Santo Domingo de los Tsáchilas in Ecuador represents the largest informal cattle market. Because of its strategic position, cattle movement is very high and therefore we selected this region, to determine the strain variation of *Brucella* sp. Part of the study aimed at the isolation, biotyping, and genotyping of *Brucella* species from milk and supra-mammary lymph nodes of sero-positive bovines, using selective Farrell medium, biochemical assays, and IS711-PCR, AMOS-PCR, and HOOF-Prints techniques. In total, 656 animals from 12 sero-positive dairy herds and from the provincial slaughterhouse were diagnosed by Rose Bengal and Wright’s Slow Agglutination test with EDTA. Amongst these animals, 50 animals were sero-positive for brucellosis. Twenty-five lymph nodes and 25 milk samples from each group of positive reactors were transferred to culture medium. Isolation was possible from 4 (16%) lymph nodes and 9 (36%) milk samples; out of these, 10 isolates were diagnosed as *Brucella* sp. All four isolates of lymphatic tissue corresponded to *Brucella abortus* biotype 1, confirmed as field strains by molecular analysis. Milk isolations, showed biochemically a more dispersed pattern in which *B. abortus* biotypes 1 and 4 were found; yet four samples gave a pattern similar to *B. abortus* biotype 2; however, only biotypes 1 and 4 were confirmed by molecular analysis. The concordance between biochemical and molecular diagnostic tests reached 76.9%.

## Introduction

Brucellosis is a widespread zoonotic disease, affecting cattle, sheep, goats, pigs, and humans (1). From a total of nine species of *Brucella* reported so far, four species are zoonoses: *Brucella abortus*, *B. canis*, *B. melitensis*, and *B. suis* which have been typically related to cattle, dogs, sheep goats, and pigs, respectively. Other species such as *B. microti*, *B. neotomae*, *B. ovis*, *B. pinipedialis*, and *B. inopinata* are supposed to be host specific (2, 3).

In cattle, the main symptoms associated with brucellosis include abortion and poor health in newborn calves. Epididymitis and infertility have been also reported in bulls (4, 5). In Ecuador, annual losses due to brucellosis in cattle are estimated to be around 5.5 million USD due to abortions, reduced milk yield, and mortality (6). In addition, in several municipalities in Ecuador, the presence of brucellosis in humans has been directly related to its presence in the cattle population (7), with, so far, only, *B. abortus* as the causative agent of human brucellosis (8, 9), contrary to neighboring Colombia and Peru, were in addition to *B. abortus*, *B. melitensis*, and *B. suis* have equally been reported in man (8, 10).

Determining the strain variability of *Brucella* can be helpful to understand the geographical and epidemiological dispersion of the disease as shown in the United States where molecular techniques have been used to evaluate strain diversity of *B. abortus* to define foci of transmission between cattle and wildlife, i.e., elk and bison, and also to identify infections related to the use of vaccines (11). In northern Ecuador, previous studies have reported *B. abortus* biotype 1 and 4 in human samples (9, 12), yet the diversity of *Brucella* sp. in cattle has not been investigated previously.

The livestock market in Santo Domingo de los Tsáchilas province is the largest in the country because of its strategic geographical location (13). This cattle market is very informal, facilitating the movement and exchange of animals and meat to large cities. It is also an important center for the trade of animals from the dairy areas of the Sierra region to different areas in the coastal region for fattening bull calves, as such it is hardly surprising that many of the outbreaks of foot-and-mouth disease started in this region (13). Thus, the sanitary condition of animals in this region might offer a reflection of the health status of cattle from different zones of the country. In this context, and given the zoonotic risk related to cattle brucellosis, the evaluation of the disease prevalence supported by a study of strain variability in cattle passing through this region will be an important epidemiological tool, including the assessment of the importance of food-borne brucellosis.

## Materials and Methods

### Study design

The study area was located at Santo Domingo de los Tsáchilas Province (0.14°: −0.70°N, −78.73°: −79.62°E). In total, 656 blood samples were collected from 12 sero-positive dairy farms, previously identified during a large-scale national survey (data not published) and at the provincial abattoir between May and June 2013. Samples were analyzed by Rose Bengal plate (RB) and Wright’s Slow Agglutination Test with EDTA (SAT-EDTA). Equally, milk and supra-mammary lymph nodes were carefully sampled avoiding contamination and stored at 4°C until screening by RB and/or SAT-EDTA. Samples from positive reactors were processed for bacterial growth in the specific growth medium.

### Serological tests

All blood samples were tested by Rose Bengal (Bengatest antigen^®^ 4% v/v suspension) and Wright’s SAT-EDTA (antigen SAW^®^, Synbiotics ASAW code). For RB, the slightest trace of agglutination was considered as positive. For SAT-EDTA, 100 μl of antigen was added to a doubling serum dilution from 1/12.5 up to 1/25.600. Data were recorded as international agglutination units (international units per milliliter) with values equal or greater than 30 IU/ml, corresponding to a transparency of 25% of a 1/25 dilution, considered as a positive reactions as described by Godfroid and Boelaert (14).

### Microbiological isolation

In a microbiology laboratory (biosafety type III), lymph nodes were macerated using the Stomacher^®^, milk samples were centrifuged at 3000 *g* for 10 min. Both macerated nodes and cream were tested for bacterial growth in selective Farrell medium [Columbia blood agar base CM0331 (Oxoid) + horse serum (reference: 16050-130 Gibco) + modified *Brucella* Selective Supplement SR0083A (Oxoid)]. Cultures were kept at 37°C and 5% CO_2_ for 5 days (15). Then, isolates were transferred to agar base [Columbia blood agar base CM0331 (Oxoid)] to obtain distinct *Brucella* sp. colonies. Finally, part of the colonies was used for DNA extraction and another part was stored at −70°C for further analysis.

### Biotyping and molecular identification

Isolated colonies were biotypified by macroscopic observation and biochemical assays, i.e., urease, catalase, oxidase, and hydrogen sulfide production. Additionally, bacterial cultures were grown on media with stained safranin, thionin, and fuchsin at different concentrations, and tested for agglutination with Anti-A and Anti-M mono-specific sera (15).

For molecular identification, genomic DNA was extracted according to Marmur and Kirby [phenol–chloroform–isoamyl alcohol (16)]. DNA amplification was performed using protocols IS711-PCR and AMOS-PCR as described by Ref. (17, 18) to identify genera and species, respectively. Primers for DNA amplification are presented in Table 1. Each PCR-reaction had a final volume of 20 μl. Master mix was made with 1 U/45 μl of Taq Polymerase, 1X buffer, 1.5 mM MgCl_2_, 0.2 mM dNTPs, 0.2 mM of each primer, and approximately 10 ng of DNA. To characterize the *Brucella* biotype, the “HOOF-Print” technique was used as described by Bricker et al. (19) and Bricker and Ewalt (20) for eight *loci*; all VNTR were amplified separately using primers described in Table 1; each PCR-reaction had a final volume of 15 μl and the master mix was composed with 0.6 U of Taq Polymerase, 1X buffer, 1.5 mM MgCl_2_, 0.25 mM dNTPs, 0.2 mM of each primer, and approximately 10 ng of DNA.

**Table 1:** **Primers used in the study**.

Primer (name)	5′-3′Sequence
**Primer sequence for IS711-PCR for genus identification**		
IS6501 3′	GAT AGA AGG CTT GAA GCT TGC GGA C
IS6501 5′	ACG CCG GTG TAT GGG AAA GGC TTT T
**Primer sequence for conventional AMOS-PCR for species identification**		
*B. abortus*-specific primer	GAC GAA CGG AAT TTT TCC AAT CCC
*B. melitensis*-specific primer	AAA TCG CGT CCT TGC TGG TCT GA
*B. ovis*-specific primer	CGG GTT CTG GCA CCA TCG TCG
*B. suis*-specific primer	GCG CGG TTT TCT GAA GGT TCA GG
IS711-specific primer	TGC CGA TCA CTT AAG GGC CTT CAT
**Primer sequence for “HOOF-prints” biotype**		**Primer (reverse)**
Locus-1	GGT GAT TGC CGC GTG GTT CCG TTG AAT GAG	REV-3
Locus-2	CCC GCA TGA TCC GCG AAC AGC TGG ATG	REV-1
Locus-3	CAG GCG CTT GAG GAT GAG GCG GCA G	REV-3
Locus-4	GCA GAA TTT TCG AGG CAT TCG GCG ATG	REV-3
Locus-5	GTG CTC CAG GGC GCC GGG AGG TAT GTT TAG	REV-3
Locus-6	GCC GCA GGA AAG CAG GCG ATC TGG AGA TTA TC	REV-3
Locus-7	CAG AGC CGT CGG TGG TTA CTT GAG TAG GGC AG	REV-1
Locus-8	GTG GGA AGC GTT ATC CTT TAA CGG GAG TAA GGG	REV-1
REV-1	GGG GAG TAT GTT TTG GTT GCG CAT GAC CGC	–
REV-3	GGG GGC ART ARG GCA GTA TGT TAA GGG AAT AGG G^a^	–

*^a^R = A to G*.

### Data analysis

The proportions of isolation of *Brucella* sp. were contrasted by Fisher exact test with 5% statistical significance. Additionally, an estimation of the test concordance was measured in terms of positive and negative agreements over the total isolations. Data were analyzed in “R” software version 3.1.0.

## Results

### Serology

Out of 656 blood samples, 50 were sero-positive, i.e., 25 were from the slaughterhouse and 25 were from sero-positive dairy farms of Santo Domingo.

### Microbiological isolation

Twenty-five milk and 25 lymph node samples were processed and isolated in a specific microbiological medium. The bacterial growth of *Brucella* spp. was evidenced in nine (36%) and four cases (16%), respectively. No statistical difference was found between the types of sample used for the isolation (*p*-value = 0.1085); yet isolation from milk appeared to be better than from tissues.

### Bio-typification

Table 2 shows the biochemical features of the microbiological isolations from sero-positive animals and from those where *Brucella* was isolated (milk or supra-mammary lymph nodes). Out of nine milk isolations, six were biochemically compatible with *B. abortus* biotype and three were “not determined” isolations (ND, samples: 8, 10, and 13) because they did not present urease activity, nor growth in CO_2_ and no H_2_S production. Isolations from lymphatic nodes (samples 1–4) were also biochemically compatible with *B. abortus*. In total, nine isolates were sensitive to inhibition by basic fuchsin, four were insensitive but agglutinated with anti-A sera. Nine isolates agglutinated with anti-A sera (i.e., samples 1–5, 6, 7, 9, and sample 11) and only one agglutinated with anti-M sera (sample 12) hence corresponding to *B. abortus* biotype 4. As described by Corbel and Brinley Morgan (21), Mayfield et al. (22), and Rodríguez Torres et al. (23), growth in basic fuchsin medium and agglutination with anti-A sera, is indicative for *B. abortus* biotype 1; however, lack of bacterial growth in basic fuchsin and agglutination with anti-A sera is indicative for *B. abortus* biotype 2. Yet, as shown in Table 2, by molecular analysis, all isolates were *B. abortus* biotype 1. All milk isolates were identified as *B. abortus* biotypes 1 and 4.

**Table 2:** **Differential characters of *B. abortus* and biotypes isolated from milk and lymph nodes collected in Santo Domingo de los Tsáchilas province**.

Sample no.	Code	Source	Activity					Growth on dye media			Agglutination in mono-specific sera		Biotype
			Oxidase	Catalase	Urease	CO_2_	H_2_S	Fuchsin	Safranin	Thionin 20 μg	Anti-A	Anti-M	
1	1482	Lymph node	**+**	**+**	**+**	**+**	**+**	**+**	**−**	**−**	**+**	**−**	*Bvar1*
2	1483	Lymph node	**+**	**+**	**+**	**+**	**+**	**+**	**+**	**−**	**+**	**−**	*Bvar1*
3	1550	Lymph node	**+**	**+**	**+**	**+**	**+**	**+**	**+**	**−**	**+**	**−**	*Bvar1*
4	1552	Lymph node	**+**	**+**	**+**	**+**	**+**	**+**	**+**	**−**	**+**	**−**	*Bvar1*
5	1476	Milk	**+**	**+**	**+**	**+**	**+**	**+**	**+**	**−**	**+**	**−**	*Bvar1*
6	1285	Milk	**+**	**+**	**+**	**+**	**+**	**−**	**−**	**−**	**+**	**−**	*Bvar2*
7	1286	Milk	**+**	**+**	**+**	**+**	**+**	**−**	**−**	**−**	**+**	**−**	*Bvar2*
8	1294	Milk	**+**	**+**	**−**	**−**	**−**	**+**	**+**	**+**	**−**	**−**	ND
9	1301	Milk	**+**	**+**	**+**	**+**	**+**	**−**	**−**	**−**	**+**	**−**	*Bvar2*
10	1302	Milk	**+**	**+**	**−**	**−**	**−**	**+**	**+**	**+**	**−**	**−**	ND
11	1306	Milk	**+**	**+**	**+**	**+**	**+**	**−**	**−**	**−**	**+**	**−**	*Bvar2*
12	1307	Milk	**+**	**+**	**+**	**+**	**+**	**+**	**+**	**−**	**−**	**+**	*Bvar4*
13	1308	Milk	**+**	**−**	**−**	**−**	**+**	**+**	**+**	**+**	**−**	**−**	ND

*ND, not determined*.

### Molecular identification

In total, 13 isolates corresponded to *B. abortus* identified by IS711 and AMOS-PCR (Table 3). The “HOOF-Prints” protocol allows biotype classification, as such VNTR markers evidenced the presence of *B. abortus* biotype 1 in 12 out of 13 isolates. All these isolates were field strains and were different from vaccine strains S19 and RB51, as confirmed by conventional AMOS-PCR. Furthermore, one isolate, from a milk sample, was confirmed to be *B. abortus* biotype 4 (Sample 12). The allelic diversity found in *Brucella* isolates from Santo Domingo Province is given in Table 4. Molecular patterns found are similar to biotype 1 and 4, reported by Bricker et al. (19). Samples, biochemically found as biotype 2 (samples 6, 7, 9, and 11), were confirmed as *B. abortus* biotype 1 whilst sample 12 was corroborated as biotype 4.

**Table 3:** **Genotyping of *Brucella* spp. from isolates of milk and lymph nodes collected in Santo Domingo de los Tsáchilas province**.

Sample no.	Code	PCR^a^-IS711	AMOS^b^-PCR	VNTR^c^
1	1482	**+**	*B. abortus*	*Bvar1*
2	1483	**+**	*B. abortus*	*Bvar1*
3	1550	**+**	*B. abortus*	*Bvar1*
4	1552	**+**	*B. abortus*	*Bvar1*
5	1476	**+**	*B. abortus*	*Bvar1*
6	1285	**+**	*B. abortus*	*Bvar1*
7	1286	**+**	*B. abortus*	*Bvar1*
8	1294	+	*B. abortus*	*Bvar1*
9	1301	+	*B. abortus*	*Bvar1*
10	1302	+	*B. abortus*	*Bvar1*
11	1306	+	*B. abortus*	*Bvar1*
12	1307	**+**	*B. abortus*	*Bvar4*
13	1308	+	*B. abortus*	*Bvar1*

*^a^PCR, polymerase chain reaction*.

*^b^AMOS-PCR, PCR for detection of *B. abortus*, *B. melitensis*, *B. ovis*, and *B. suis**.

*^c^VNTR, variable number of tandem repeat*.

**Table 4:** **HOOF-Prints: results of alleles configuration to identify *Brucella abortus* biotypes from isolates of milk collected in Santo Domingo Province in Ecuador**.

Sample^a^	Code	Locus-1	Locus-2	Locus-3	Locus-4	Locus-5	Locus-6	Locus-7	Locus-8	Biotype
6	1285	4	3	6	6	5	6	3	2	Bvar1
7	1286	4	3	6	6	5	6	3	2	Bvar1
9	1301	4	3	6	6	5	6	3	2	Bvar1
11	1306	4	3	6	6	5	6	3	2	Bvar1
12	1307	7	4	5	3	2	2	7	2	Bvar4

*^a^Samples shown in this table correspond to samples that were different from B. abortus Bvar1 in biotyping; i.e., Biotype 2 and 4*.

On the other hand, the concordance of biochemical and molecular tests estimated a proportion of coincidences of 76.92%.

## Discussion

This study demonstrated the presence of bovine brucellosis in the province of Santo Domingo de los Tsáchilas province.

Biochemical tests used for biotyping isolates allowed the identification of *B. abortus* biotypes 1, 2, and 4, biotypes which have been previously reported in human populations in Ecuador using biochemical and molecular techniques (9, 12). Samples 6, 7, 9, and 11, were biochemically identified as *B. abortus* biotype 2, yet as *B. abortus* biotype 1 by HOOF-Prints protocol, which is highly sensitive test (11, 19). It is known that the biochemical tests are of limited use for identifying biotypes, since the biochemical response depends on environmental conditions during the preparation of media and reagents and the amount and time for growth of the strains (24–26). In addition, the intraspecific *Brucella* molecular variability could have caused this biochemical response (21–23, 27). However, further studies are suggested to confirm or reject the presence of *B. abortus* biotype 2 in Ecuador or that the biochemical results are due to a genetic adaptation of *B. abortus* biovar 1.

Molecular tests indicated that all strains described in this study were field strains and not vaccine-type strains; as for *B. abortus* biotype 1 field strains, in spite of being genetically similar to vaccine strains, the former do not grow in thionin (2 μg/ml) in a culture medium.

The presence of *B. abortus* biotype 4 as previously reported in humans by Ron-Román et al. (9), was confirmed in this study. The biochemical characteristics of *B. abortus* biotype 4 differ from *B. abortus* biotype 1 and 2 because the former is agglutinated by anti-A instead of anti-M sera. In the same way, the allelic configuration allowed differentiating between biotypes 1 and 4 in HOOF-Prints technics.

The type strains of all classical *Brucella* species and biovars were surveyed to assess the discriminating power of microsatellite fingerprint technique. This technique was used to assess the level of divergence amongst and within populations of naturally infected cattle and wildlife (19, 20, 28, 29).

In this survey, both *B. abortus* biotype 1 and 4 were reported as described by Ron-Román et al. (9, 12) in humans from northern Ecuador. The presence of the two biotypes (1 and 4) in animals in Santo Domingo province shows that due to intensive cattle movement, the presence of several biotypes is possible. Finally, the study findings suggest that microbiological isolation of *Brucella* spp. is more successful from milk samples (44%) than from lymph nodes in slaughter cattle (16%).

In conclusion, the strain diversity of *B. abortus* was assessed in a region with intensive cattle movement and *B. abortus* biotypes 1 and 4 were found; although, some isolations of *B. abortus* biotype 1 presented phenotypic variability according to biochemical tests. These findings were correlated with results found in humans in northern Ecuador. Further research is needed to study intra-species variability and to investigate the possibility of other biotypes and *Brucella* species present in the tropical regions of Ecuador.

## Conflict of Interest Statement

The authors declare that the research was conducted in the absence of any commercial or financial relationships that could be construed as a potential conflict of interest.

## References

[1] Acha P Szyfres B “Brucellosis” in Zoonoses and Communicable Disease Common to Man and Animals. Third ed (Vol. 1). Scientific and Technical Publication No. 580. Washington, DC: Pan America Health Organization (2001). p. 40–67.

[2] Sauret JM Vilissova N Human brucellosis. J Am Board Fam Pract (2002) 15(5):401–6. 12350062

[3] Scholz HC Nöckler K Göllner C Bahn P Vergnaud G Tomaso H *Brucella inopinata* sp. nov., isolated from a breast implant infection. Int J Syst Evol Microbiol (2010) 60:801–8. 10.1099/ijs.0.011148-0 19661515

[4] England T Kelly L Jones R MacMillan A Wooldrige M. A simulation model of brucellosis spread in British cattle under several testing regimes. Prev Vet Med (2004) 63:63–73. 10.1016/j.prevetmed.2004.01.009 15099717

[5] Yamamoto T Toshiyuki T Nishiguchi A Kobayashi S. Evaluation of surveillance strategies for bovine brucellosis in Japan using a simulation model. Prev Vet Med (2008) 86:57–74. 10.1016/j.prevetmed.2008.03.004 18440660

[6] Agrocalidad. Programa Nacional de Control de la Brucelosis Bovina. (2009). Available from: http://www.agrocalidad.gob.ec/agrocalidad/images/pdfs/sanidadanimal/programa_nacional_brucelosis_bovina.pdf

[7] Ron L Benítez W Speybroeck N Ron J Saegerman C Berkvens D Spatio-temporal clusters of incident human brucellosis cases in Ecuador. Spat Spatiotemporal Epidemiol (2013) 5:1–10. 10.1016/j.sste.2013.02.001 23725883

[8] Lucero NE Ayala SM Escobar GI Jacob NR. *Brucella* isolated in humans and animals in Latin America from 1968 to 2006. Epidemiol Infect (2008) 136:496–503. 10.1017/S0950268807008795 17559694 PMC2870831

[9] Ron-Román J Ron-Garrido L Abatih E Celi-Erazo M Vizcaíno-Ordóñez L Calva-Pacheco J Human brucellosis in Northwest Ecuador: typifying *Brucella* spp., seroprevalence, and associated risk factors. Vector Borne Zoonotic Dis. (2014) 14(2):1–10. 10.1089/vbz.2012.1191 24410144

[10] Tique V Daza E Álvarez J Mattar S Seroprevalencia de *Brucella abortus* y ocurrencia de *Brucella melitensis* en caprinos y ovinos de Cesar y Sucre. Revista UDCA Actual de Divulgación Científica (2010) 13(2):133–9.

[11] Higgins J Stuber T Quance C Edwards W Tiller R Linfield T Molecular epidemiology of *Brucella abortus* isolates from cattle, elk and bison in the United States, 1998 to 2011. Appl Environ Microbiol (2012) 78:3674–84. 10.1128/AEM.00045-12 22427502 PMC3346378

[12] Ron-Román J Saegerman C Minda E Benítez W Brandt J Douce R. Case report; first report of orchitis in man caused by *Brucella abortus* biovar 1 in Ecuador. Am J Trop Med Hyg. (2012) 87:534–8. 10.4269/ajtmh.2012.11-0341 22826490 PMC3435359

[13] Lindholm A Hewitt E Torres P Lasso M Echeverria C Shaw J Epidemiologic aspects of a foot-and-mouth disease epidemic in cattle in Ecuador. Int J Appl Res Vet Med (2007) 5(1):17–24.

[14] Godfroid J Boelaert F Prescriptions Pour le Diagnostic Serologique de la Brucellose. Belgium: CODA-CERVA (Ed.) (1995). 47 p.

[15] Alton G Jones L Angus R Verger J Techniques for the Brucellosis Laboratory. Paris: INRA (1988).

[16] Surzycki S Basic Techniques in Molecular Biology. Berlin: Springer Editorial (2000). p. 1–31.

[17] Bricker B Halling S. Differentiation of *Brucella abortus* bv. 1, 2, and 4, *Brucella melitensis*, *Brucella ovis*, and *Brucella suis* bv. 1 by PCR. J Clin Microbiol (1994) 32:2660–6. 7852552 10.1128/jcm.32.11.2660-2666.1994 PMC264138

[18] Bricker B Halling S. Enhancement of the *Brucella* AMOS PCR assay for differentiation of *Brucella abortus* vaccine strains S19 and RB51. J Clin Microbiol (1995) 33:1640–2. 7650203 10.1128/jcm.33.6.1640-1642.1995 PMC228233

[19] Bricker B Ewalt D Halling S. *Brucella* ‘HOOF-Prints’: strain typing by multi-locus analysis of variable number tandem repeats (VNTRs). BMC Microbiol (2003) 3:15. 10.1186/1471-2180-3-15 12857351 PMC183870

[20] Bricker B Ewalt D. Evaluation of the HOOF-print assay for typing *Brucella abortus* strains isolated from cattle in the United States: results with four performance criteria. BMC Microbiol (2005) 5:37. 10.1186/1471-2180-5-37 15975142 PMC1183211

[21] Corbel MJ Brinley Morgan WJ Proposal for minimal standards for description of new species and biotypes of the genus *Brucella*. Int J Syst Bacteriol (1975) 25(1):83–9 10.1099/00207713-25-1-83

[22] Mayfield J Bantle J Ewalt D Meador V Tabatabai L Detection of *Brucella* cells and cell components. In: Nielsen K Duncan R editors. Animal Brucellosis. Florida: CRC Press (1992). p. 97–101.

[23] Rodríguez Torres A Abad R Orduña A Especies y biovars del género *Brucella*. Etiología de la brucelosis humana en España. Enfermedades Infecciosas y Microbiología Clínica (1992) 10:43–8. 1498175

[24] Saegerman C Berkvens D Godfroid J Walravens K Bovine brucellosis. In: Lefévre P Blancou J Chermette R Uilenberg G editors. Infectious and Parasitic Diseases of Livestock. London: Lavoisier (2011). p. 991–1021.

[25] Godfroid J Nielsen K Saegerman C. Diagnosis of brucellosis in livestock and wildlife. Croat Med J (2010) 51:296–305. 10.3325/cmj.2010.51.296 20718082 PMC2931434

[26] Godoy M Orozco L Identificación de micobacterias no tuberculosas: comparación de métodos bioquímicos y moleculares. Revista de la Sociedad Venezolana de Microbiología (2011) 28:96–104.

[27] Minharro S Silva JP Dorneles EMS Pauletti RB Neubauer H. Biotyping and genotyping (MLVA16) of *Brucella abortus* isolated from cattle in Brazil, 1977 to 2008. PLoS One (2013) 8(12):e81152. 10.1371/journal.pone.0081152 24324670 PMC3855697

[28] Wang Y Chen C Cui B Liu J. [Comparative study on identity of *B. ovis* 019 strain by traditional methods and HOOF-prints technique]. Wei Sheng Wu Xue Bao (2007) 47(2):240–3. 17552227

[29] Kang S-I Her M Heo EJ Nam HM Jung SC Cho D. Molecular typing for epidemiological evaluation of *Brucella abortus* and *Brucella canis* isolated in Korea. J Microbiol Methods (2009) 78(2):144–9. 10.1016/j.mimet.2009.05.009 19463862

